# Periodontitis and vascular inflammatory biomarkers: an experimental in vivo study in rats

**DOI:** 10.1007/s10266-019-00461-3

**Published:** 2019-10-03

**Authors:** Yago Leira, Ramón Iglesias-Rey, Noemí Gómez-Lado, Pablo Aguiar, Tomás Sobrino, Francesco D’Aiuto, José Castillo, Juan Blanco, Francisco Campos

**Affiliations:** 1 Periodontology Unit, Faculty of Medicine and Odontology, University of Santiago de Compostela, Medical-Surgical Dentistry (OMEQUI) Research Group, Health Research Institute of Santiago de Compostela (IDIS), Santiago de Compostela, Spain; 2 Periodontology Unit, UCL Eastman Dental Institute and Hospital, University College London, 256 Gray’s Inn Road, London, WC1X 8LD UK; 3 Clinical Neurosciences Research Laboratory, Clinical University Hospital, Health Research Institute of Santiago de Compostela (IDIS), Travesa da Choupana s/n, 15706 Santiago de Compostela, Spain; 4 Molecular Imaging Group, Clinical University Hospital, Faculty of Medicine, University of Santiago de Compostela, Health Research Institute of Santiago de Compostela (IDIS), Santiago de Compostela, Spain

**Keywords:** Periodontitis, Lipopolysaccharide, *Porphyromonas gingivalis*, Inflammation, Endothelial dysfunction

## Abstract

The objective of this preclinical in vivo study was to determine changes in vascular inflammatory biomarkers in systemic circulation after injection of lipopolysaccharide (LPS) from *Porphyromonas gingivalis* (*Pg*) in rats. Experimental periodontitis was induced by injections of *Pg*-LPS. Gingival soft and hard tissues changes were analysed by means of magnetic resonance imaging and micro computed tomography. Serum levels of interleukin (IL)-6, IL-10, pentraxin (PTX) 3, and soluble fragment of tumor necrosis factor-like weak inducer of apoptosis (sTWEAK) were determined at baseline and 24 h, 7, 14, and 21 days after periodontal induction. Significant periodontal inflammation and alveolar bone loss were evident at the end of periodontal induction. Experimental periodontitis posed an acute systemic inflammatory response with increased serum levels of IL-6 and PTX3 at 24 h post-induction, followed by a significant overexpression of sTWEAK at 7 days. This inflammatory state was maintained until the end of the experiment (21 days). As expected, IL-10 serum levels were significantly lower during the follow-up compared to baseline concentrations. In the present animal model, experimental periodontitis is associated with increased systemic inflammation. Further studies are needed to confirm whether PTX3 and sTWEAK could be useful biomarkers to investigate potential mechanisms underlying the relationship between periodontitis and atherosclerotic vascular diseases.

## Introduction

Atherosclerotic vascular diseases (AVDs) such as coronary heart disease and ischemic stroke have been associated with periodontitis [1, 2]. Although the potential mechanisms behind this relationship have not been fully elucidated, it has been hypothesized that microorganisms such as *Porphyromonas gingivalis* (*Pg*) and their products [e.g., lipopolysaccharides (LPS)] can access to peripheral blood circulation invading vascular endothelial cells [3] and evoking acute-phase reactions with elevated levels of circulating pro-inflammatory cytokines [4]. Other hypothetical scenario would be that locally produced pro-inflammatory mediators within the gingiva could be dumped into the systemic circulation promoting vascular changes in the endothelium, thus, contributing to atheroma plaque formation [4, 5].

Besides interleukin-6 (IL-6) that induces hepatic synthesis of pentraxins (PTXs) such as C-reactive protein or serum amyloid P component, increased circulating levels of non-induced hepatic PTX3 have been associated with the presence of AVDs and higher risk of mortality [6, 7]. In patients with cardiovascular disease plasma PTX3 is negatively correlated with flow-mediated dilatation (FMD), a hallmark of endothelial dysfunction [8].

Tumor necrosis factor-like weak inducer of apoptosis (TWEAK), a member of the tumor necrosis factor superfamily of cytokines, can be released with biological activity as soluble TWEAK (sTWEAK) [9]. TWEAK is expressed in several tissues (i.e., heart, brain, and lung) and cells (endothelial and smooth muscle cells). Human studies showed that in patients with chronic kidney disease, sTWEAK was associated with impaired FMD [10, 11] and atheromatosis progression [12]. It was also found that in artery samples from patients with atherosclerosis, sTWEAK concentrations were correlated with increased carotid intima-media thickness (c-IMT), a hallmark of subclinical atherosclerotic disease [13].

Gingival tissues of periodontal patients showed increased levels of PTX3 and sTWEAK compared to non-periodontally affected tissues [14, 15]. However, little is known regarding the potential effect of experimental periodontitis on serum levels of these two mediators that are related to vascular inflammation. Therefore, we aimed to test the hypothesis that LPS-induced periodontitis could contribute to peripheral changes of inflammatory biomarkers (IL-6, IL-10, PTX3, and sTWEAK) in rats.

## Materials and methods

### Animal experiments and study design

An experimental in vivo study was carried out in the Clinical Neurosciences Research Laboratory of the University Clinical Hospital of Santiago de Compostela (REGA ES 15078 029 2801). All experimental procedures were performed according to the Animal Care Committee European Union rules and the Spanish regulation (86/609/CEE, 2003/65/CE, 2010/63/EU, RD1201/2005 and RD53/2013). The Animal Research Reporting of In Vivo Experiments guidelines were followed in this experiment [16].

The experimental design was recently described elsewhere [17]. Briefly, six systemically healthy male Sprague–Dawley rats of 7 weeks of age and weighing between 300 and 350 g were used. Animals were housed individually, in stable environmental conditions (environmental temperature of 23 °C), relative humidity of 40% and a light–dark cycle of 12 h, as well as free access to food and water. Each animal was initially placed into an induction chamber attached to a sevoflurane anaesthetic vaporizer and anaesthesia was induced with 6% sevoflurane in a gas of 70% NO_2_ and 30% O_2_, followed by the application of a nose cone with 4% sevoflurane in the same proportion of the aforementioned gases to maintain anaesthesia during the experimental procedures. During surgery, all animals were subjected to temperature control, maintaining temperature at 37 ± 0.5 °C by a thermostat-controlled electric pad (NeoBiotect, Spain).

### *Porphyromonas gingivalis* lipopolysaccharide-induced periodontitis

Aliquots of 10 μL were prepared adding 1 mL of homogenized endotoxin-free water to 1 mg LPS from *Pg* (lyophilized). In total, 27 aliquots were prepared and stored at 4 °C, of which 24 were needed for the purpose of the study. The palatal gingiva between the first and second maxillary molars in both sides (i.e., right and left sides) was injected with 10 μL of a saline solution containing 1 mg/mL LPS from *Pg* (tlrl-pglps, InvivoGen, San Diego, CA) using a Hamilton microsyringe (Agilent, Santa Clara, CA, USA) equipped with a blunted edge 30-gauge needle. This injection was followed by two additional injections at 48-h intervals as described previously [17, 18]. This protocol was repeated the following week as recommended by the previous publications [17, 19]. Therefore, periodontitis induction lasted 14 days with a total of 6 injections of *Pg*-LPS per side. To have a good access to perform the injections, the animals were placed facing up and the mouth was maintained open with a special microsurgical separator.

All experiments were performed by a single surgeon/periodontist (YL).

### Magnetic resonance imaging (MRI) analysis

All animals were anesthetized by inhalation of 5% sevoflurane in an NO_2_/O_2_ mixture (70/30). Rectal temperature was maintained at 37 ± 0.5 °C using a feedback-controlled heating pad.

#### MRI examination

All samples were analysed using a 9.4 T horizontal bore magnet (Bruker BioSpin, Ettlingen, Germany) with 440 mT/m gradients and a combination of a linear birdcage resonator (7 cm in diameter) for a signal transmission and a 2 × 2 surface coil array for signal detection.

#### MRI assessment

MR mouth localizer images in axial and coronal orientations were determined to then position accurately the slices of the molars to study, corresponding to rapid acquisition with relaxation enhancement (RARE) T2 sequence: with an echo time (EcT) = 11.7 ms, repetition time (RT) = 2 s, rare factor = 4, flip angle (FA) = 180°, number of averages (NA) = 1, spectral bandwidth (SW) = 50 kHz, 10 slices of 1 mm, 25 × 25 mm^2^ field of view (FOV) (with saturation bands to suppress signal outside this FOV), a matrix size of 192 × 192 (isotropic in-plane resolution of 130 μm/pixel × 130 μm/pixel) and implemented without fat suppression option.

The progression of the area with periodontal inflammation was determined from T_2_-weighted (T_2_-w) and T_1_-weighted (T_1_-w) images acquired 0, 7, and 14 days after the onset of periodontal induction. Axial T_1_-w were obtained with the use of rapid acquisition with relaxation enhancement with variable repetition time (RAREVTR) sequence: with an EcT = 8.44 ms, RT = 12 s, rare factor = 4, FA = 180°, NA = 3, SW = 50 kHz, 20 slices of 0.5 mm, 19.2 × 19.2 mm^2^ FOV (with saturation bands to suppress signal outside this FOV), a matrix side of 192 × 192 (isotropic in-plane resolution of 100 μm/pixel × 100 μm/pixel) and implemented without fat suppression option. Axial T_2_-w were obtained with the use of RARE T2 sequence: with an EcT = 12.66 ms, RT = 2 s, rare factor = 4, FA = 114°, NA = 6, SW = 50 kHz, 20 slices of 0.5 mm, 30 × 30 mm^2^ FOV (with saturation bands to suppress signal outside this FOV), a matrix side of 256 × 256 (isotropic in-plane resolution of 117 μm/pixel × 117 μm/pixel) and implemented without fat suppression option.

#### Data processing

**Fig. 1:**
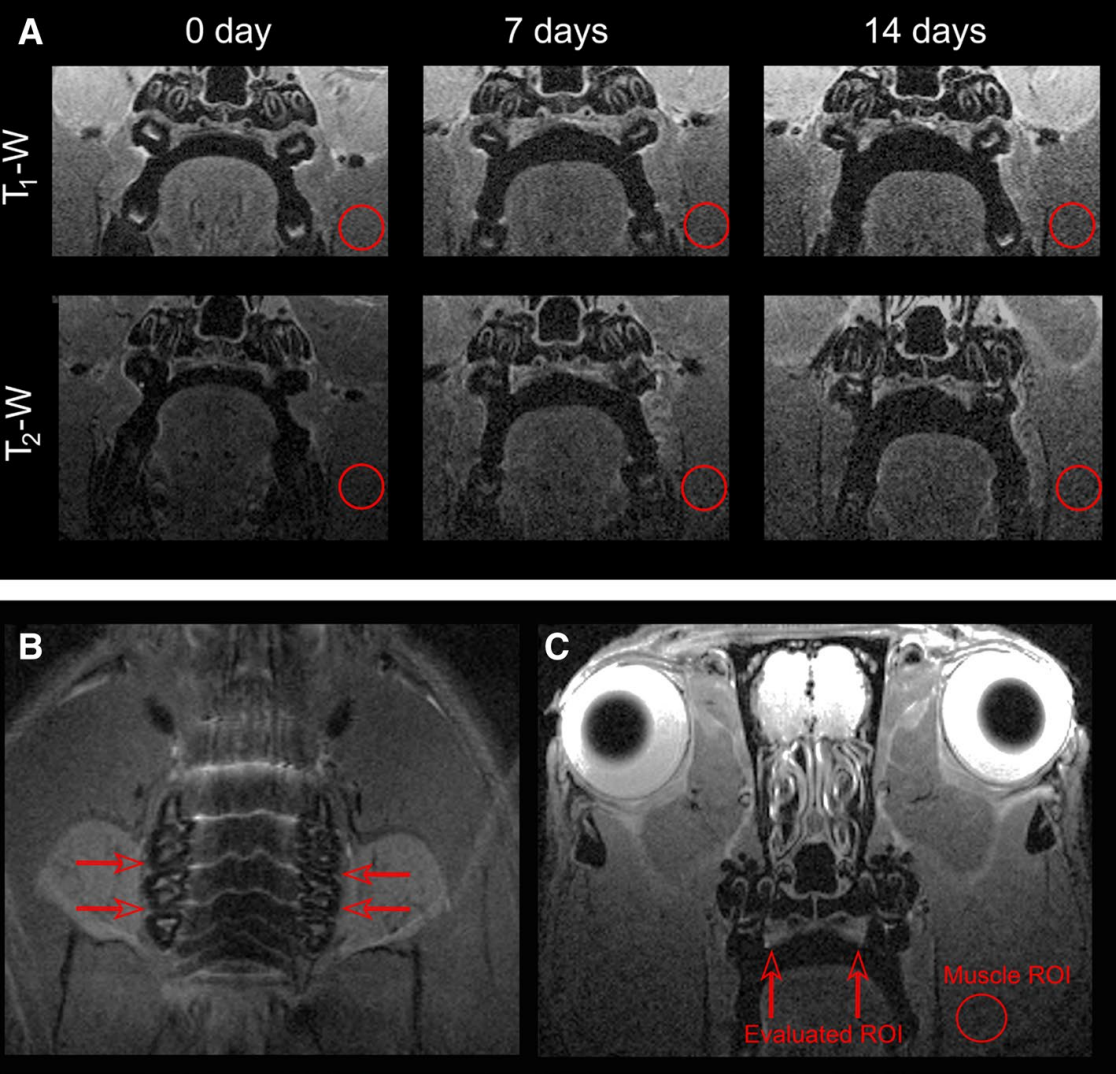
**a** T_1_-w and T_2_-w images of periodontal inflammation evolution affecting the first upper molars. **b** Coronal MR localizer image. In order to locate the same plane every week, red arrows indicate the planes of study. **c** Representative axial T_2_-w weighted image. Red arrows show the position of the molars evaluated. The location of the region of interest (red circle) was used to measure the muscle signal intensity (color figure online)

Images were processed using ImageJ (Rasband WS, National Institutes of Health, Bethesda, MD, USA) on an independent computer workstation. From the T_1_-w and T_2_-w images, the signal intensity of the gingival palatal mucosa within the molar region was studied (from mesial to distal of the first and second upper molars) (Fig. 1a). To focus accurately in the same plane of the mouth every week, planes were adjusted taking into account the localizer images (Fig. 1b, c).

Because absolute changes in MR signal intensity cannot readily be compared between individuals, we normalized these parameters and expressed them as relative changes in signal intensity. Relative changes in signal intensity of the regions of interest (ROIs) were calculated by dividing the mean signal intensity changes by the respective muscle ROI signal, with the resulting number expressed as a percentage [signal of intensity = (ROI mean)/(muscle ROI mean) × 100] [20].

Location and dimension of the area with periodontal inflammation were determined from T_1_-w images as a relative gingival palatal thickness respect to the onset of the periodontal induction. The average using six values per molar and side (i.e., first and second molars both right and left) for T_1_-w, T_2_-w, and palatal thickness measurements was used for the analysis.

All MRI analyses were performed by a single physicist (RI-R).

### Micro-computed tomography (μCT) analysis

All animals were anesthetized by inhalation of 5% sevoflurane in an NO_2_/O_2_ mixture (70/30) using a bed centered in the head of the animal. Rectal temperature was maintained at 37 ± 0.5 °C using a feedback-controlled heating pad.

#### μCT examination

Maxillae were scanned using a μCT scanner (Bruker BioSpin, Woodbridge, Connecticut, USA) with a voxel size of 0.045 mm (isotropic voxel) and X-ray energy of 45 kV and 400 μA. Each scan was conducted over a period of 35 min at 0 and 14 days.

#### Alveolar bone loss measurement

To quantify de amount of bone loss, the bone level was measured at the sagittal plane of both sides of each animal. Crossing the interproximal contact point of the first and second molars crown, the distance between de cemento-enamel junction (CEJ) and the alveolar bone crest were measured for the distal surface of the first molar and the mesial surface of the second molar just below the contact point and 0.2 mm palatal to the contact point [21]. The average using the six measurements per side (right and left separately) was used for the analysis.

All μCT analyses were performed by a single biologist (NG-L).

### Serum collection and laboratory tests

Prior to periodontitis induction (baseline) and 24 h, 7, 14, and 21 days after the last series of injections, 1800 μL of venous blood were collected from the tail of each animal by venepuncture using a 22-gauge needle with a 1 mL syringe. Blood samples were allowed to clot at room temperature, and after 1 h, serum was separated from blood by centrifugation (7 min at 3000*g*) and 700 μL of extracted serum was immediately transferred to 1.5 mL Eppendorf tubes. Each tube was stored at − 80 °C until the time of analysis. Serum levels of all biomarkers were measured by enzyme-linked immunosorbent assay (ELISA) technique following manufacturer instructions. IL-6 ELISA kit (PicokineTM, Boster Biological Technology, Pleasanton, California, USA) minimum assay sensitivity was 5.0 pg/mL with a intra-assay coefficient of variation of 1.7%; IL-10 ELISA kit (PicokineTM, Boster Biological Technology, Pleasanton, California, USA) minimum assay sensitivity was 4.0 pg/mL with a intra-assay coefficient of variation of 7.4%; PTX3 ELISA kit (Fine Test, Wuhan Fine Biotech, Wuhan, China) minimum assay sensitivity was 0.094 ng/mL, with a intra-assay coefficient of variation of 1.5%; sTWEAK ELISA kit (Fine Test, Wuhan Fine Biotech, Wuhan, China) minimum assay sensitivity was 9.375 pg/mL, with a intra-assay coefficient of variation of 4.4%. Determinations were performed in the Clinical Neurosciences Research Laboratory.

### Statistical analysis

All data analyses were performed with IBM SPSS Statistics 20.0 software for Mac (SPSS Inc., Chicago, IL, USA). Mean and standard deviation was calculated for continuous variables, after the method of Shapiro–Wilk was applied to confirm that the data were sampled from a normal distribution. Paired *t* test was used to compare differences in terms of alveolar bone loss between baseline and 14 days. Changes over time in MRI measurements as well as biomarkers were assessed with repeated measure analysis of variance. In addition, post hoc comparisons were carried out using Bonferroni corrections. Pearson correlation coefficient (*r*) was used to assess potential correlations between serum biomarkers levels and alveolar bone loss.

All tests were performed at a significance level of *α* = 0.05.

## Results

### Periodontal inflammation

Clinical inflammation within the gingiva was evident at day 7 of the experiment affecting the palatal side of the first and second molar regions in both sides of the upper jaw.

**Fig. 2:**
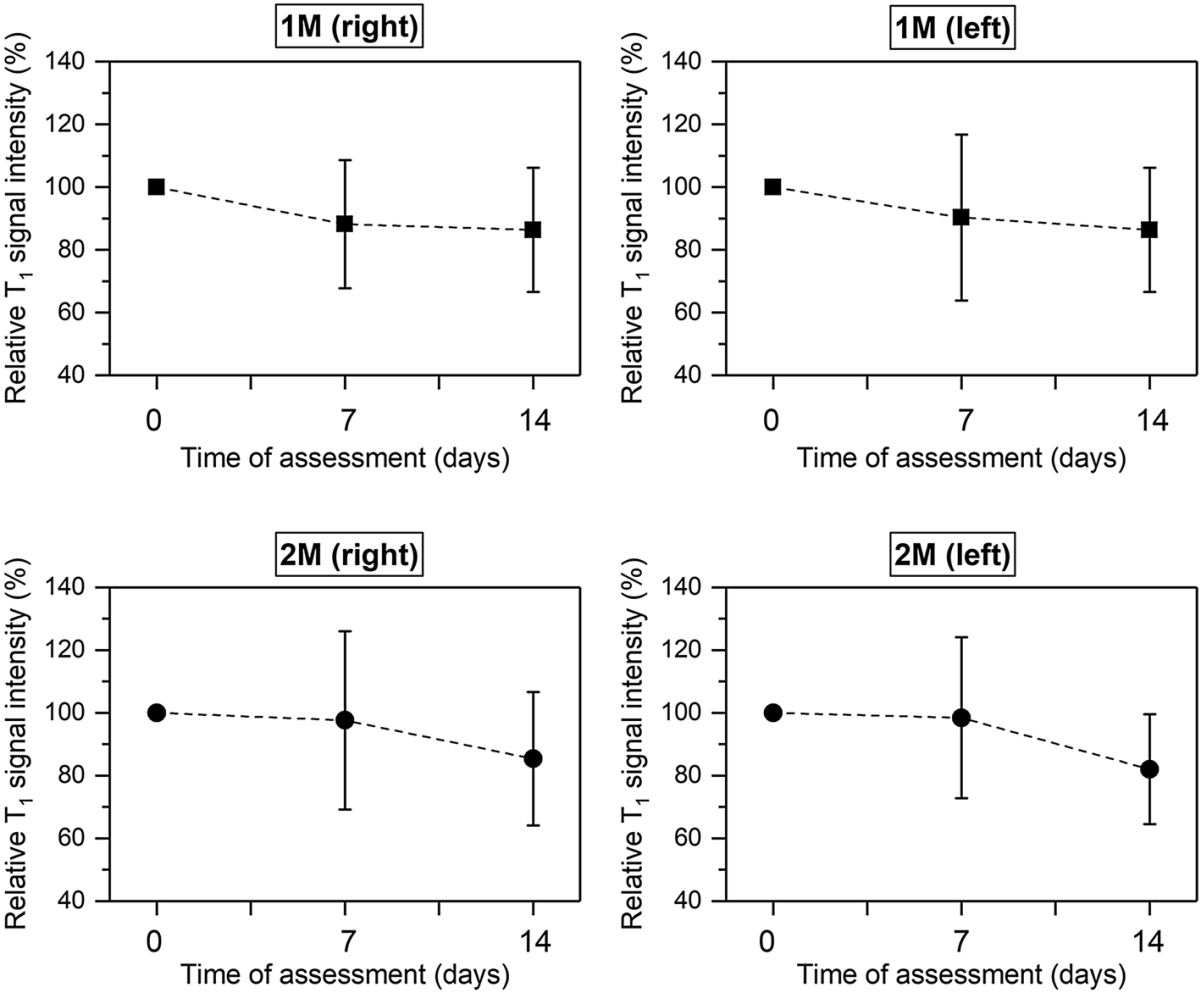
Percentage of relative T_1_-w signal intensity at baseline, 7 and 14 days of experiment measured in first and second upper molars both from the right and left side. Black dots and squares represent mean values and bars correspond to plus/minus standard deviation. No significant differences were found between time points

**Fig. 3:**
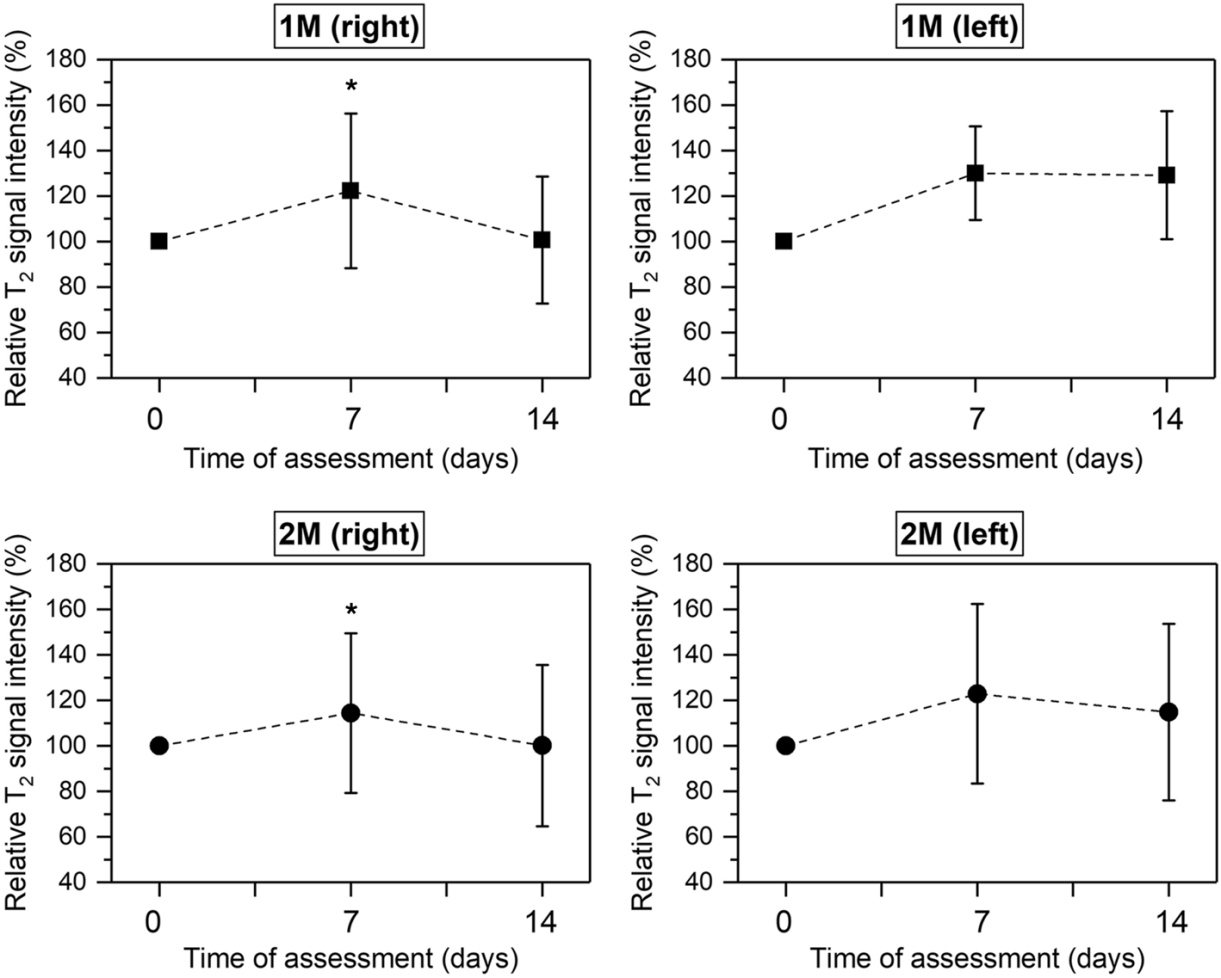
Percentage of relative T_2_-w signal intensity at baseline, 7 and 14 days of experiment measured in first and second upper molars both from the right and left sides. Black dots and squares represent mean values and bars correspond to plus/minus standard deviation. *Significant differences between 7 and 14 days (*P* < 0.05)

Periodontal inflammation was confirmed by means of MRI analysis. Differences in relative T_1_ and T_2_ signal intensities were found at 7 and 14 days after periodontal induction onset compared to baseline (Figs. 2, 3). When relative T_2_-w signal intensity was analysed in the first and second upper right molar, statistical differences were observed between 7 and 14 days (*P* < 0.05).

**Fig. 4:**
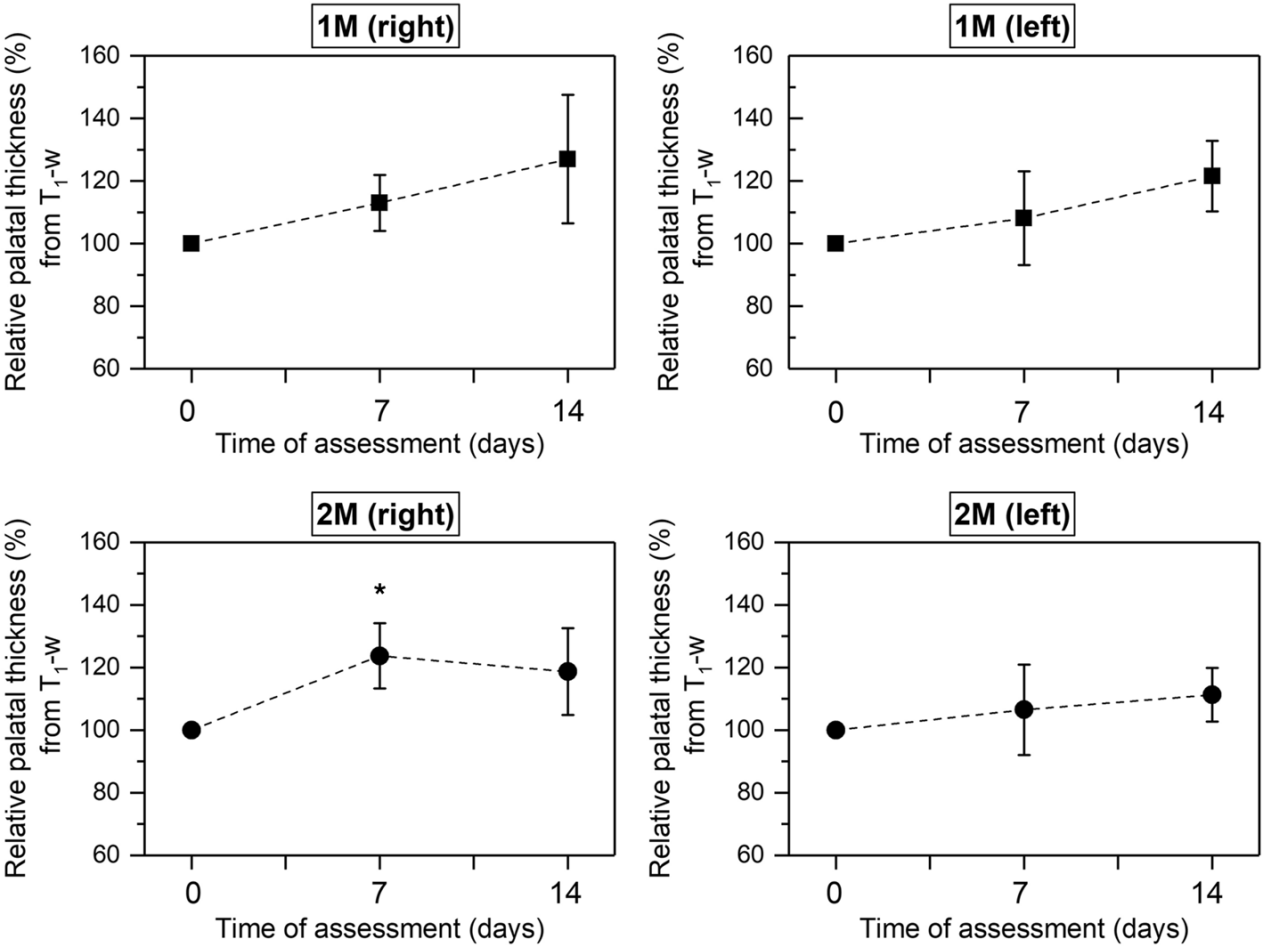
Percentage of relative palatal thickness from T_1_-w at baseline, 7 and 14 days of experiment measured in first and second upper molars both from the right and left sides. Black dots and squares represent mean values and bars correspond to plus/minus standard deviation. *Significant differences between 0 and 7 days (*P* < 0.01)

In terms of gingival palatal thickness, which we considered as a surrogate measure of oedema, an increase in the relative percentage was found after the induction of LPS-induced experimental periodontitis. In fact, statistically significant differences were observed for this measure in the second upper right molar between 7 days of periodontal induction and baseline (Fig. 4).

### Alveolar bone loss

**Fig. 5:**
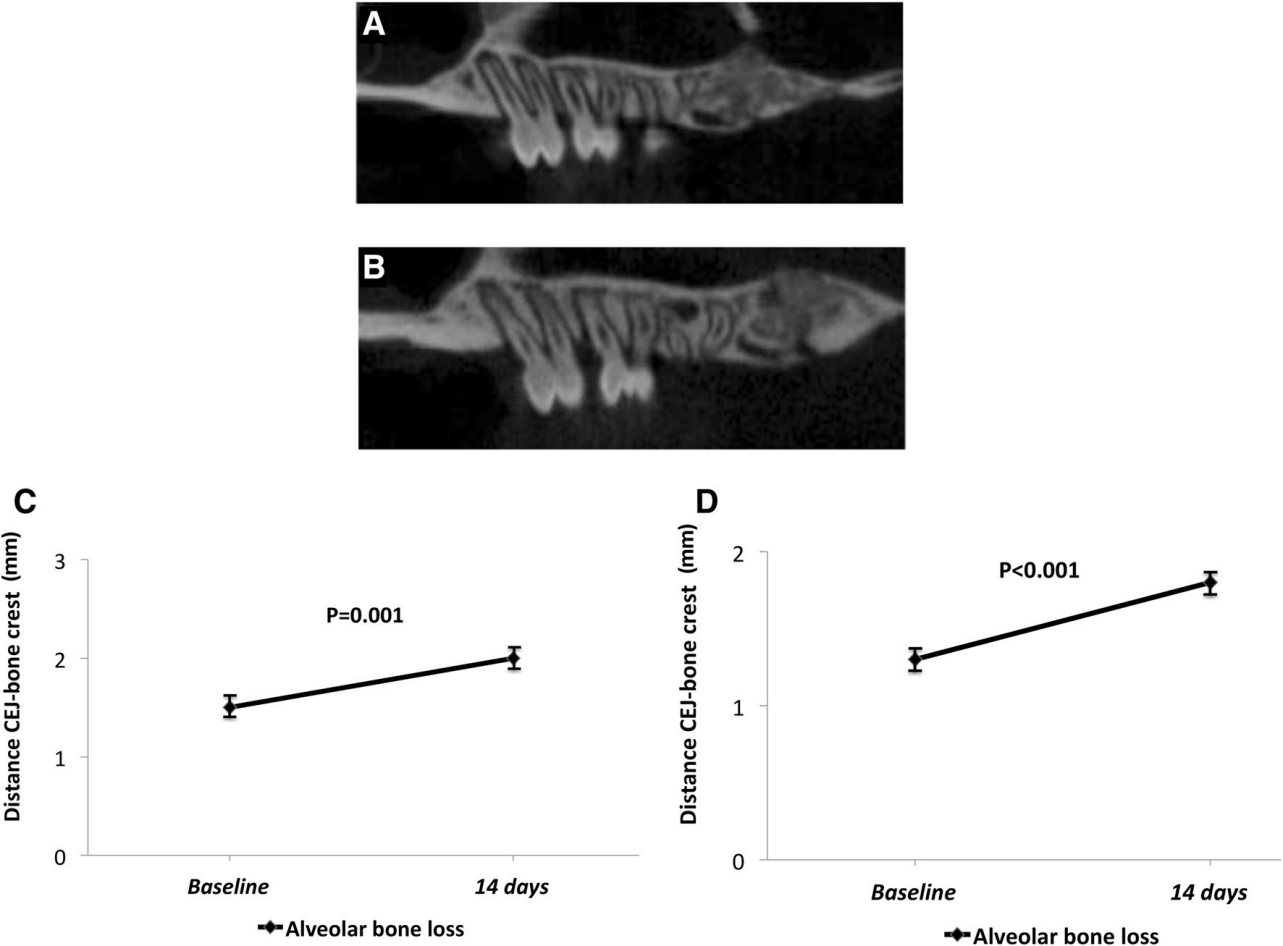
**a** μCT image of upper molars at baseline. **b** μCT image of upper molars at 14 days where it can be appreciated bone loss produced by LPS-induced periodontitis. **c** Alveolar bone loss in the upper right side. The distance between the CEJ and the bone crest was significantly greater 14 days after the first LPS injection (*P* = 0.001). **d** Alveolar bone loss in the upper left side. Significant differences in the distance from the CEJ to the alveolar bone crest at 14 days of experiment were observed (*P* < 0.001)

The μCT analysis revealed statistical significant alveolar bone loss at the interproximal space after periodontal induction (14 days) between the first and second maxillary molars bilaterally at the LPS-injected sites compared to baseline (Fig. 5a, b). Indeed, the distance between the cemento-enamel junction and the bone crest was significantly greater at 14 days in both sides of the upper jaw compared to baseline measurements (Fig. 5c, d).

### Biomarkers

**Fig. 6:**
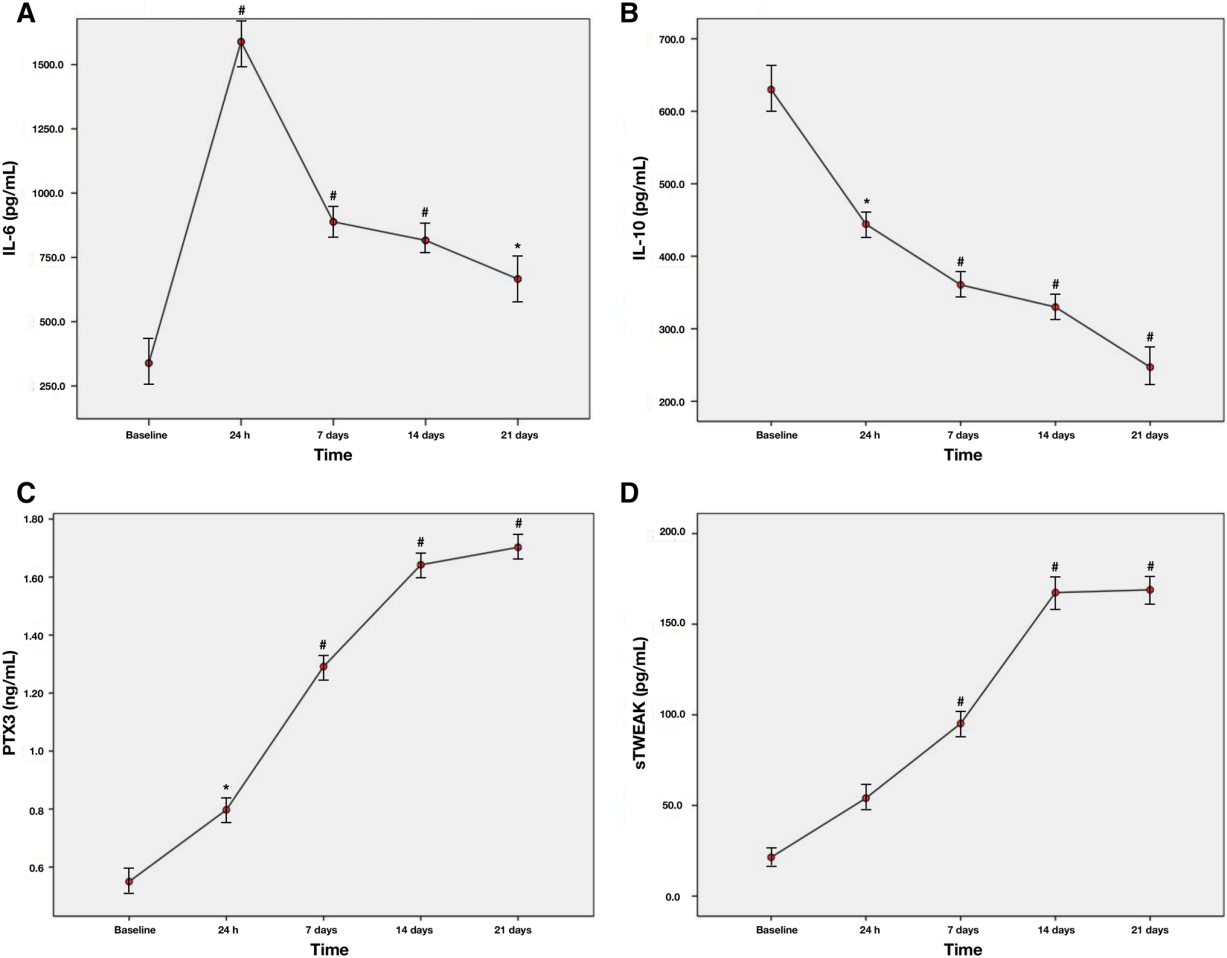
Changes in serum levels of: **a** IL-6 (pg/mL); **b** IL-10 (pg/mL); **c** PTX3 (ng/mL); **d** sTWEAK (pg/mL) at 24 h, 7, 14, and 21 days after periodontal induction. Red dots represent mean values and bars correspond to plus/minus standard deviation. ^#^Significant differences compared to baseline for serum levels of biomarkers (*P* < 0.001), *Significant differences compared to baseline for serum levels of biomarkers (*P* < 0.05)

A sharp increase was observed for IL-6 24 h after periodontal induction. The levels of IL-6 decreased during the following 3 weeks, but still were significantly higher compared to baseline (Fig. 6a).

On contrary, periodontitis evoked a significant decrease in IL-10 serum levels at 24 h after the last LPS injection and continued to reduce up to 21 days (Fig. 6b).

A mild acute significant increase in PTX3 levels at 24 h following periodontal induction was observed, which was more pronounced as time was drawn on (Fig. 6c). Levels of sTWEAK were significantly elevated 1 week after the last LPS-injection compared to baseline and continued to increase in the following weeks (Fig. 6d).

### Correlation between biomarkers and alveolar bone loss

We found a positive correlation between mean alveolar bone loss at 14 days and serum levels of IL-6 at 24 h (*r* = 0.905, *P* = 0.013), sTWEAK at 14 and 21 days (*r* = 0.896, *P* = 0.016; *r* = 0.932, *P* = 0.007) as well as with PTX3 at 7, 14 and 21 days post-induction (*r* = 0.835, *P* = 0.039; *r* = 0.971, *P* = 0.001; *r* = 0.921, *P* = 0.009).

## Discussion

The relationship between inflammation and AVDs is supported by the high presence of inflammatory cells within the plaque tissue and the circulating levels of inflammatory mediators [4]. In the present animal study, experimental periodontitis was associated with increased serum levels of systemic inflammatory biomarkers such as IL-6, PTX3, and sTWEAK during 21 days of experiment.

Here, we showed an elevation of serum IL-6 in rats with periodontitis, which was more evident right after the induction process (24 h). Accordingly, we found a positive correlation between alveolar bone loss assessed by μCT and serum levels of IL-6 at 24 h. Our results are similar to those in showed in previous animal and human studies, in which increased serum levels of IL-6 were associated with the presence of periodontitis [22–26]. There is currently evidence that IL-6 plays an important role in propagating the inflammatory response that is responsible for atherosclerosis [27]. Stimulation of hepatic synthesis of acute-phase reactants, activation of endothelial cells and increased coagulation are some of the main effects exerted by this cytokine. On contrary, we found that serum levels of the anti-inflammatory mediator IL-10 were significantly lower after periodontal induction, thus, confirming that periodontitis may predispose to a systemic inflammatory state.

PTX3 has also been studied as a contributor to vascular endothelial dysfunction. In the rodent model, exogenous administration of PTX3 significantly blunted nitric oxide production through the matrix metalloproteinase (MMP)-1 and P-selectin pathway leading to morphological alterations of endothelial cells [28]. In addition, there is clinical evidence that plasma PTX3 is negatively correlated with FMD [8]. We observed that serum levels of PTX3 were significantly elevated 24 h after periodontal induction, and remained increased up to 21 days. Furthermore, alveolar bone loss positively correlated with IL-6 levels at 7 and 14 days as well as 21 days post-induction. These results are in accordance with those obtained by Keles and co-workers, in which they found that ligature-induced periodontitis was associated with increased serum levels of PTX3 and this biomarker was correlated with alveolar bone loss either in the group of experimental periodontitis with 10 or 40 days of periodontal induction [29]. Studies carried out in chronic periodontal patients have shown a positive correlation between PTX3 levels and periodontal clinical parameters such al clinical attachment level (a measure of prolonged exposure to periodontitis) and probing pocket depth (an indicator of current periodontitis) at both local [30] and systemic levels [31].

TWEAK/Fn14 axis also showed some effects on monocytes and macrophages involved in the inflammatory process of atheromatosis. Through Fn14 inhibition, the interaction TWEAK/Fn14 may alter macrophage trafficking and increase lipid uptake of macrophages [32]. In addition, TWEAK was found to induce several pro-inflammatory mediators of atherogenesis such as IL-6, monocyte chemotactic protein-1, and IL-8 in activated monocytes [33] as well as able to enhance MMP-9 and enhance MMP-2 activity in apoe E null mice [34]. Clinical evidence suggested that in chronic kidney disease patients, there is a relationship between sTWEAK levels and FMD [10, 11], subclinical atherosclerosis [13] as well as atheromatosis progression [12]. In our study, sTWEAK serum levels were significantly increased 7 days after periodontitis induction and remained elevated until the end of the experiment. Alveolar bone loss was correlated with the levels of sTWEAK at 14 and 21 day post-induction. Evidence suggested that gingival tissues of periodontal patients overexpress TWEAK and the TWEAK/Fn14 axis could be responsible for the induction of IL-1β, intracellular adhesion molecule-1 and vascular cell adhesion molecule-1 within human gingival fibroblasts, which are molecules involved in the dysfunction of the vascular endothelium [35, 36].

Recently, periodontitis was found to be associated with high levels of IL-6, PTX3, and sTWEAK in patients with cerebral small vessel disease increasing almost 3 times the likelihood of having this type of AVD [37]. If our experimental findings are replicated in humans, patients with periodontitis should undergo periodontal therapy to treat their gums and to reduce the systemic inflammatory state linked with this disease, which in turn could prevent periodontal patients for being at a high risk for having vascular events.

We have to be cautious when interpreting our results because of the low number of animals included in our analysis (*N* = 6) and due to some limitations inherent to the experiment design. Even though the lack of a negative control group, vehicle group or even a sham-operated group could be one of the major criticisms, we considered the biomarker levels at baseline of each animal as a control per se due to our purpose was to analyse the changes of each biomarker after injections of one of the bacterium that has been associated with periodontal infection. Similarly, a recent animal study with the same experimental design also considered baseline values of the molecules as a control [17]. Furthermore, experimental data showed that the systemic inflammatory state of a control group remains similar over time with no significant changes on the levels of IL-6 (e.g., 15 and 30 days after the start of the experiment) [38]. Another important issue is the animal model used in the present study. The main limitation of LPS model is the lack of bacterial colonization present in human periodontitis and the need for constant injections throughout the experiment. In addition, periodontal infection is not due to one single pathogen; therefore, the translational significance of this model could be limited. For example, ligature-induced model allows plaque accumulation in the gingival margin similar to what happens in humans but placing the ligatures around teeth could produce mechanical lesions that may aggravate the periodontal destruction [39, 40]. Similarly, multiple injections can also produce trauma on the gingiva leading to inflammatory reactions that may mask true periodontal inflammation. In fact, if only evaluated clinically or by MRI, we cannot ensure that periodontitis is induced. However, bone loss (a hallmark of periodontitis) was detected by μCT. In our experiment, clinical examination was done only visually (e.g., redness, swelling, spontaneous bleeding, etc) without a validated method. On contrary, radiographic bone loss was measure according to previous studies using a validated and reproducible method [17, 21]. Although bearing in mind some of the aforementioned shortcomings of the LPS model, it is considered to be a direct and easy method for the induction of controlled periodontitis bone loss is localized, the stimulus is constant, and the alveolar bone destruction usually occurs within 7 days after the start of the bacterial LPS injections [41]. Furthermore, this model is useful to investigate the host-bacteria interaction and the activation of signalling pathways, pro-inflammatory and vascular dysfunction mediators as well as performance of specific cells or molecules in the pathogenic process of periodontitis [42]. Therefore, we considered this model as the most suitable to test our hypothesis. Finally, there are some limitations regarding animal models that tend to overestimate findings compared to human research. Specifically in this case, for instance, age and sex of the animals used (often to study diseases that are found in the elderly with other comorbidities and in which the gender could play a role in the development or progression of the disease), different immune systems that lead to a different immune response, or the use of an acute model to develop a chronic condition [43].

In conclusion, our preliminary results showed increased serum levels of IL-6, PTX3, and sTWEAK after *Pg*-LPS injections. Further experimental studies are warranted to confirm our findings and to investigate possible mechanisms through which periodontal inflammation could contribute to the overexpression of sTWEAK and PTX3. Human studies should be carried out to assess whether in periodontal patients, PTX3 and sTWEAK are related to c-IMT and FMD. If so, interventional trials would be the next step to test whether periodontal treatment could lower circulating levels of these biomarkers and, therefore, have a beneficial impact on vascular health in periodontal patients with other comorbidities.
